# The Art and Science of Thyroid Surgery in the Age of Genomics: 100 years after Theodor Kocher

**DOI:** 10.4274/2017.26.suppl.01

**Published:** 2017-01-09

**Authors:** Seza Gulec

**Affiliations:** 1 Florida International University Herbert Wertheim College of Medicine, Departments of Surgery and Nuclear Medicine, Miami, USA

**Keywords:** Genomics, thyroid cancer, thyroidectomy, radioactive iodine, beta knife

## Abstract

Cancer is a disorder of the genome. The thyroid cancer genome is being decoded. Recent studies have identified a mutation or a genetic alteration in 95% of thyroid cancers. The National Cancer Institute initiated the Cancer Genome Atlas project in 2006 to catalogue genetic mutations associated with cancer, using genome sequencing and bioinformatics. The project has expanded to carry out genomic characterization and sequence analysis of thyroid cancer. The concept of risk stratification based on traditional parameters will soon vacate their role for clear molecular markers of non-invasive/focal, invasive/metastatic and systemic stages/phases of neoplastic disorder. A refined classification scheme based on genomics and its phenotypic expressions will accurately reflect the biologic differences between the different morphologic definitions we use today. Tumor differentiation/de-differentiation, and clinical behavior of an individual cancer will be defined by molecular markers, in addition to standard morpho-pathology. Empiricism in science of medicine and surgery has acquired a new method for testing the appropriate treatment for individual patients; that is molecular pathology, governed by genomics. The technology is present and rapidly evolving. The surgeons will determine the extent of interventions with molecular evidence and guidance.

## Abstract (tr)

Tiroid cerrahisinin bilimsel temelleri Theodor Kocher tarafından tanımlandı. Bu ustayı 100 yıl önce uğurladık. Tirod kanseri cerrahisi ve tiroid kanseri hastalarının tedavisi ile tartışmalar süregelmektedir. Konunun mihengi açıkçası ilk aşama cerrahi tedavinin boyutudur. Cerrahi tedaviyi izleyen kısa ve uzun dönemde uygulanabilecek tedaviler, yararlanılabilecek tetkik ve görüntüleme yöntemleri de ilk cerrahi tedavinin çerçevesiyle alakalıdır. Amerikan Tiroid Birliği’nin 2015 basımı tedavi kılavuzu dahil olmak üzere, geleneksel olarak tiroid kanserinin klinik değerlendirmesi morfolojik öğelere dayalı yapılır. Tümör büyüklüğü, histolojik varyantlar ve hasta yaşı kriterleri cerrahi ve cerrahi sonrası girişimlerin boyutunu belirler. Kanser genomu projesi kapsamında tiroid kanseri genom profili aydınlanmaya başladı. Hala birçok bilinmeyen olmakla birlikte bu konuda bilgi birikimimiz hızla artıyor. İnce iğne biyopsisi materyalinde hastaların kromozom değişiklikleri ve nokta mutasyonları tespit edilebilmekte, bu genomik değişikliklerin biyolojik anlamları araştırılabiliyor. Bu teknoloji hızla teşhis ve tedavi yönlendirilmesinde kullanıma giriyor. Bundan böyle tedavi seçimlerinde genomik kılavuz ve yönlendirme kullanılacak. Bu makale cerrahi sanatının mahir ustalarını ve cerrahi bilimindeki genomik devrimi selamlıyor.

The extirpation of the thyroid gland typifies, perhaps better than any operation, the supreme triumph of the surgeon’s art. A feat which today can be accomplished by any competent operator without danger of mishap and which was conceived more than one thousand years ago might appear an unlikely competitor for a place in surgery so exalted.

**William Stewart Halsted**

## Milestones

**Figure 1:**
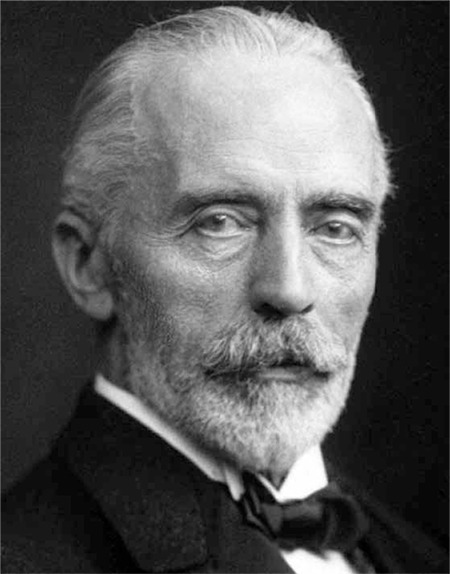
Theodor Kocher

Theodor Kocher died 100 years ago in 1917. Kocher is considered the father of thyroid surgery (Figure 1).

He received the 1909 Nobel Prize in Physiology or Medicine for his work in the physiology, pathology and surgery of the thyroid. A few weeks before his death, at the age of 76, he made his final appearance before the Swiss Surgical Congress, reviewing his entire thyroid surgery experience. Kocher reported on approximately 5.000 operations with a mortality of about 0.5%. When he started his work in the 1870s, thyroid surgery was a high-risk procedure, with an estimated mortality of 75% in 1872. Thyroid operations were prohibited by the Academy of Medicine in France at that time. Kocher was appointed to the Chair of Surgery in Berne, Switzerland, in 1872, at the age of 31, and began his influential work in thyroid surgery and medicine. His most acclaimed achievement, his surgical technique, was marked by meticulous care in dissecting and ligating blood vessels, and precise dissection within the thyroid capsule (1). William Halsted’s impression on Kocher’s surgical technique was/is quite remarkable. Halsted described Kocher’s technique as “neat and precise, operating in a relatively bloodless manner, scrupulously removing the entire thyroid gland doing little damage outside its capsule” (2). Kocher was the first to describe the devastating complication(s) of total extirpation of the thyroid gland. He also recognized the underlying pathophysiologic changes in a diseased gland. This was, in a sense, a “molecular vision.” He intuitively contemplated that the growth of goiter nodules was an early determined event in altered thyroid physiology, and that the abnormal thyroid tissue was the source of a goiter recurrence. He conceived the notion of autonomously growing, focally distributed clusters of follicular cells in nodular goiter. He, thus, advocated a total thyroidectomy rather than selective removal of all thyroid nodules. Kocher had realized that the so-called “subtotal” thyroidectomy, leaving behind naturally growth-prone tissue, would lead to goiter recurrence. The concept was called “Innere Chirurgie” (internal surgery), a scientific surgical philosophy based on biological considerations. This was the beginning of a new epoch. The leading minds were Kocher in Berne, Halsted in Baltimore, and Mikulicz (coined the term) in Krakau (3).

Despite impressive discoveries in surgical anatomy and physiology, the onco-biology of neoplasia and the clinico-pathologic characteristics of cancer were poorly understood at Kocher’s time. Although Kocher had significant studies on malignant tumors of the thyroid gland, the modern science for cancer surgery was developed by William Halsted, who championed a radical treatment approach for breast cancer. Halsted’s idea was based on the premise that cancer had a linear, step-wise growth fate and had to be be treated with total extirpation of the organ along with its lymphatic drainage network. This was believed to be necessary for cure, and was adapted by many prominent surgeons. This classic rationalistic philosophy in surgery dominated the surgical world for years. Similarly, thyroid surgery for cancer in the early post-Kocher era, called for an “en-block resection” or “conventional radical neck dissection,” which usually sacrificed the sternocleidomastoid muscle, internal jugular vein, often the accessory nerve and sometimes the marginal mandibular branch of the facial nerve. This was all justified in the name of “cure. The core problem with the rationalistic logical flow is the potential/possible flaw in the original hypothesis. The entire chain of thoughts/deductions, then, may lead to incorrect conclusions (a-priori error). The outcomes, however, need to be tested independently. (post-priori validation). This is the essence of evidence-based, data-driving scientific methodology”. George “Barney” Crile Jr. should be credited as the first to challenge the radical thought process and action. He was one of the first surgeons ever to promote the idea that “the less surgery the better,” and he campaigned vigorously for the abandonment of the radical operations. His ideas on thyroid cancer surgery, and later breast cancer surgery, were briskly opposed at the time, but eventually succeeded (4). The new surgical vision was reluctantly, but progressively accepted by the surgical community over time, and has since, shaped the evolution of the philosophy of surgical treatment.

Mid-century brought in a major innovation in the management of thyroid cancer, the radioactive iodine (RAI) treatment. First used by Samuel Seidlin (5), and established as a fortitude by Bierwaltes (6), RAI became an invaluable theranostic agent. The role for RAI in the management of metastatic differentiated thyroid cancer (DTC) became indisputable. Its utilization post-operatively, however, is still a matter of debate. As a guide to perplexed; RAI treatment is given in 3 distinct settings with distinct clinical indications (intents). 1) Ablation of the remnant 2) Adjuvant treatment for residual disease or occult metastatic disease 3) Therapy for known metastatic disease. The term “ablation” specifically refers to first-line RAI treatment following total surgical thyroidectomy. The specific target of this treatment is normal residual thyroid tissue- the remnant. The objectives of ablation are three-folds a) Ablation eradicates all the functioning thyroid tissue. Thereby, thyroglobulin (Tg) becomes a highly specific tumor marker. This simply facilitates the post-op long-term follow-up. b) Ablation wipes out all focal normal thyroid tissue left in-situ by the surgeon to avoid injury to laryngeal nerves and parathyroid glands. From surgical standpoint these small clusters of normal tissue remnants are inconsequential, however, they appear as focal areas of RAI uptake on future whole body imaging studies and can easily be called as metastatic disease. Eradication of these potential source of misdiagnosis, when surgeon to imager communication is still on-line, is important. c) Post-ablation whole-body scan is an excellent extent of disease evaluation tool. When the post-operative RAI treatment is contemplated with an adjuvant intent, in addition to the ablation objectives, RAI is aimed to target residual disease or occult metastatic disease. When the intent is adjuvant treatment, the risk stratification becomes important in the selection of administered activity of I-131. For remnant ablation purposes only, the risk stratification has no bearing. The therapeutic effect of I-131 works through the beta particles, thus, it should be referred as “beta-knife.” Complete thyroidectomy, in the strictest sense is only possible with surgical (cold steel knife) thyroidectomy, followed by I-131 ablation (beta knife) (Table 1).

The second half of the 20th century also witnessed the birth of neo-empiricism in the acquisition and application of scientific knowledge in medicine. The established dogmas for radical surgical treatments were challenged, asking for proof of efficacy based on outcome data. The new paradigm was most palpable in the management of breast cancer and thyroid cancer. The radical versus conservative surgery argument was relatively easy to settle for breast cancer. Bernard Fisher in the US and Umberto Veronesi in Italy ran parallel trials resulting in clear demonstration of equivalency of lesser surgery over radical operations. The problem with thyroid cancer, however, remained unsettled. DTC was/is a more indolent cancer with a much more protracted course. Clinical trials with required statistical power were very difficult to perform. The lack of definitive clinical trials unleashed a never-ending controversy: Total thyroidectomy versus less than total thyroidectomy. A new, rather ambiguous, lexis for such operations entered into common use. Sub-total, near-total thyroidectomy terms subsisted. At least for the sake of clarification of the nomenclature, there should only be two standard defining operations: Total thyroidectomy and lobectomy. Total thyroidectomy is defined as the safe removal of the thyroid gland with oncologically clean margins. If/when eradication of all functioning thyroid tissue is the end point, a cold steel knife is followed by the beta-knife.

## The Equipoise

An equipoise is a genuine disagreement among the experts as to the optimal therapeutic approach in the management of a particular condition. A true equipoise exists in the initial treatment of DTC. There are two camps, represented by two diametrically opposing philosophical thoughts. There are those aggressively favor/defend the strategy of total thyroidectomy with radioiodine ablation and periodic Tg screening for “biochemical evidence of recurrence”. On the opposite direction there are those taking a conservative stand and prefer/defend performing thyroid lobectomy, when the tumor is small and limited to one lobe of the gland. This stance would automatically rule out post-surgical ablation as well as “affect” the utility of Tg screening. This approach relies more on clinical and ultrasound findings for “clinical evidence of recurrence.” It is a true equipoise, as there is genuine uncertainty in the expert medical community over which approach is more beneficial. The option of lobectomy (pertaining to tumors measuring 1cm or less), was proposed in the 2009 American Thyroid Association (ATA) guidelines (7). It became the recommended option for small tumors in its 2015 edition (8). A more progressive conservatism is endorsed in the 2010 version of the Japanese guidelines for the treatment of thyroid tumors. In the Japanese guidelines, the indications for a lobectomy were extended to tumors as large as 4 cm, if they are limited to one lobe of the thyroid with little or no extra-capsular invasion and no gross lymph node involvement. As for papillary carcinoma less than 1cm, some Japanese surgeons are proposing that observation without surgical intervention may be sufficient (9).

**The Japanese Guidelines, 2010**

A) It is beyond dispute that patients with the following characteristics are regarded as high-risk; tumor size >5 cm, lymph node metastasis >3 cm, lymph node metastasis extending to the internal jugular vein, carotid artery, major nerves such as recurrent laryngeal nerve, and prevertebral fascia, multiple and intensely swollen lymph node metastasis, extrathyroidal extension to the trachea and esophageal mucosa and distant metastasis at diagnosis. Total thyroidectomy is recommended for patients having one or more of these characteristics (p.108)

B) Other patients are classified as a “gray-zone”, but in these patients total thyroidectomy is recommended if the tumor size is >4 cm, and clinical node metastasis is detected (regardless of whether it is N1a or N1b) (p.108)

C) Although further studies with larger patient numbers and longer follow-up times are required, observation without immediate surgery for papillary microcarcinoma without metastasis or invasion can be considered a reasonable option (p.121).

It would have been easier to compose a narrative if the history of thyroid surgery was a linear progression from a radical operation towards a lesser one. The story, however, is more confounded, partly due to the particular characteristics of the disease and perhaps more so due to entrenched positions of opinionated surgeons and oncologists, the very definition of equipoise. All respectable institutions, and the thought leaders in the field have made their contributions to the controversy (10,11,12,13). Equipoise has become the standard of care in the initial surgical treatment of thyroid cancer.

Authors on both sides of the debate have pointed out that not all papillary carcinomas are equally indolent. Some grow rapidly and progress more aggressively than others often without obvious histological differences. Histological variations such as the tall cell variant and columnar cell variant have been identified, but many unusually malignant strains cannot be morphologically distinguished as being different from other examples of papillary carcinoma. Certain well-DTC will go on to have an aggressive course. They cannot be histopathologically differentiated from those with typical indolent course There appears not to have an exact way of identifying those relatively infrequent differentiated cancers that are destined to have a more malignant course. Despite the significant progress in molecular pathology, the clinical risk factors are currently the only stratification guide used in thyroid cancer diagnosis and management. Proponents of total thyroidectomy insist that the increase in complications is minimal in the hands of an experienced surgeon. Proponents of lobectomy point out that only one recurrent laryngeal nerve is at risk when only one lobe of the thyroid is being resected, thus the theoretical risk of nerve palsy is halved. Disagreement over the merits of prophylactic central node dissection has been argued in a similar context. Experience suggests that the incidence of surgical complications may not solely be dependent on the proficiency of the surgeon, but also the extent of surgical procedure performed. So the debate goes on, “equipoetically”.

## The American Thyroid Association Guidelines, 2015

The 2015 ATA guidelines is a 133 page document written in a dissertation format. The rationale for each recommendation was discussed, in detail, in a scholarly fashion. The section on operative approach for a biopsy diagnostic for follicular cell-derived malignancy (B7, Recommendation 35) defines three categories. 

The guideline committee states that in properly selected low to intermediate risk patients, the extent of initial thyroid surgery probably has little impact on disease specific survival. While recurrence rates can be quite low in properly selected patients, it is likely that the lowest rates of recurrence during long term follow-up would be associated with a total thyroidectomy. However, since salvage therapy would be quite effective in the few patients that recur after

**American Thyroid Association 2015 Guidelines**

A) For patients with thyroid cancer >4 cm, or with gross extra-thyroidal extension (clinical T4), or clinically apparent metastatic disease to nodes (clinical N1) or distant sites (clinical M1), the initial surgical procedure should include a near-total or total thyroidectomy and gross removal of all primary tumor unless there are contraindications to this procedure. (Strong Recommendation, Moderate-quality evidence),

B) For patients with thyroid cancer >1 cm and <4 cm without extra-thyroidal extension, and without clinical evidence of any lymph node metastases (cN0), the initial surgical procedure can be either a bilateral procedure (near-total or total thyroidectomy) or a unilateral procedure (lobectomy). Thyroid lobectomy alone may be sufficient initial treatment for low risk papillary and follicular carcinomas; however, the treatment team may choose total thyroidectomy to enable RAI therapy or to enhance follow-up based upon disease features and/or patient preferences. (Strong Recommendation, Moderate-quality evidence),

C) If surgery is chosen for patients with thyroid cancer <1 cm without extra-thyroidal extension and cN0, the initial surgical procedure should be a thyroid lobectomy unless there are clear indications to remove the contralateral lobe. Thyroid lobectomy alone is sufficient treatment for small, unifocal, intra-thyroidal carcinomas in the absence of prior head and neck irradiation, familial thyroid carcinoma, or clinically detectable cervical nodal metastases (Strong Recommendation, Moderate-quality evidence).

thyroid lobectomy, a conservative management approach to up front surgery accepting a slightly higher risk of loco-regional recurrence is an acceptable management strategy.

The guideline committee further stated that a more selective use of RAI coupled with a greater reliance on neck ultrasound and serial serum Tg measurements for detection of recurrent disease is likely to significantly decrease the mandate for total thyroidectomies in low and intermediate risk patients done solely to facilitate RAI remnant ablation and follow-up. Near-total or total thyroidectomy is necessary if the overall strategy is to include RAI therapy postoperatively, and thus is recommended if the primary thyroid carcinoma is >4 cm, there is gross extra-thyroidal extension, or regional or distant metastases are present. For tumors that are between 1 and 4 cm in size, either a bilateral thyroidectomy (total or near-total) or a unilateral procedure (thyroid lobectomy) may serve as the surgical platform for an overall treatment plan. Older age (>45 years), contralateral thyroid nodules, a personal history of radiation therapy to the head and neck, or familial DTC may be criteria for recommending a bilateral procedure. This could help the plans for RAI therapy, facilitate follow-up strategies, and also may address suspicions of bilateral disease (8). This conclusion returns us back to square one. The accurate identification of individual patients who would benefit from a limited intervention versus those who would require the full artillery in hand remains challenged. Clearly, a statistically insignificant, but clinically very important group of outliers of risk categories defined using standard criteria does exist. Particularly for DTC, morphology (or size)-alone only partially explains the full onco-biology of the tumor.

## Risk Stratification and Staging of Thyroid Cancer

All cancers present as either localized or at an advanced/metastatic stage, as does the thyroid cancer. The existing paradigm equates metastatic stage to a systemic disorder. The established staging system has become ingrained in our culture. Some of the seemingly “self-evident” concepts require revision. The extent of disease has been defined by the TNM system, and stages have been determined by outcome statistics. For DTC, patients younger than 45, are assigned to stage II despite the presence of remote metastatic disease (Table 2). A 2016 consensus report based on a review of 9484 patients from 10 institutions proposed a change in the cutoff age in the current American Joint Committee on Cancer/Union for International Cancer Control staging system from 45 years to 55 years. This change is estimated to lead to a down-staging of 12% of patients, and would improve the statistical validity of the existing model. Such a change may be clinically relevant for thousands of patients worldwide by preventing over-staging of patients with low-risk disease while providing a more realistic estimate of prognosis for those who remain high risk (14). The current paradigm has been inadequate to resolve the growing controversies. There does not appear to be a magic age cut-off that dicotomizes the patients into distinct risk categories. A remote metastatic disease may not be the ultimate grave sign. There may be another “phase” in cancer, besides the T, N, and M extensions, that has not been factored-in current disease state definitions. There could be a metabolic switch converting cancer into a “systemic disorder”. There may be a difference between a widespread disease and a systemic disorder, which would define/explore the cancer-host metabolic interactions beyond mere expansion of the cancer compartment. The metabolic and catabolic changes associated with this change in phase have not been identified. The road to fatality could be a phase change during any given stage of cancer expansion. The phase change could well be occurring in M0 disease or, adversely develop at a seemingly early stage. The cellular/subcellular dynamics of a true systemic disorder remain undefined. The predicative molecular signatures of the phase change has not been firmly defined. Ultimately, the molecular profiling of individual cancers would be gauging the credence of our knives. The foreseeable paradigm change awaits the maturation of the genomics revolution.

## The Age of Genomics

Cancer is a disorder of the genome.

Cancer is a disorder of the genome. The complete genetic information of an organism is referred to as genome. In all-encompassing terms, the genome involves the genes (coding and the non-coding sequences of the DNA), RNA(s), the process of transcription and translation and their regulations. The concept of regulation involves the genetic constituents such as enhancer, promoter, transcription factors (TF), RNA polymerase and mechanisms such as epigenetics. Cancer is a complex expression of multiple genetic and epigenetic alterations. There is an initiating transformational mutational event, then a multitude of genetic aberrations compose a cancer phenotype. All cells throughout the body bear the same coding sequence, yet, tissues and organs have distinct morphology and functions. Normal tissue/organ differentiation is simply a function of selective reading of the genome by different cell types, most vivid in the developing embryo. Cancer is a distinct differentiation, or neo-differentiation, or dedifferentiation, in reference to the normal tissue morphology/function. As cells enter into a constitutive act of replication or repeated cycles of growth and division, the central hallmark of cancer, they also begin to assume distinct phenotypes. This is the process of neo-differentiation. The cells undergoing the neo-differentiation process become committed to form different type of tissue rather than the mature, conformal normal tissue. The genome reading has a new translation.

The gene expression is modulated by “the switches”. The orchestration of gene expression by “the switches” involves multiple elements; The enhancer, a non-coding DNA sequence where the TF bind. The binding of the TF is arbitrated by a group of mediator proteins. The enhancer sequence is located some distance upstream of the promotor region, another non-coding DNA sequence, to which RNA polymerase binds. All these elements are also product of different set of coding genes (subject to mutational changes). Moreover, if a particular switch is deemed a compartment, the intra-compartmental interactions are subject to the chemical conformations altered by acetylation or methylation reactions which are not mutational, but referred to as “epigenetic” changes.

The thyroid cancer genome is being decoded. Recent studies have identified a mutation or a genetic alteration in 95% of thyroid cancers. The National Cancer Institute initiated the Cancer Genome Atlas (TCGA) project in 2006 to catalogue genetic mutations associated with cancer, using genome sequencing and bioinformatics. The project has expanded to carry out genomic characterization and sequence analysis of a multitude of cancers, including thyroid cancer. Techniques involve gene expression profiling, copy number variation profiling, single nucleotide pleomorphism genotyping, genome wide DNA methylation profiling, microRNA profiling, and exon sequencing of at least 1,200 genes. TCGA is able to sequence the entire genomes of tumors, including at least 6,000 candidate genes and microRNA sequences. 

The Integrated Genomic Characterization of Papillary Thyroid Carcinoma (IGCPTC) study, the first systematic, large scale and robust genomic analysis of thyroid cancer, was published in October 2014 (15). The IGCPTC study demonstrated the role of somatic genetic “alterations.” The driving somatic genetic alterations included SSNVs, indels, or fusions, in the mitogen-activated protein kinases (MAPK) and PI3K pathways in PTC. The relatively low overall density of somatic mutations was concluded to be the biological basis for the indolent clinical behavior of PTC. It is evident that the mutations and alterations are functions of time to accompany the aging process. The prognostic importance of age is a continuous variable, not determined by a cut-off value. New driver mutations were identified in PTC, either entirely novel (EIF1AX), or novel alterations of known drivers (RET, BRAF and ALK fusions) (Figure 1). As a result of these discoveries, the “dark matter” of the PTC genome has been reduced substantially from ∼25% to less than 4%, which will have profound consequences for preoperative cancer diagnosis of thyroid nodules as well as for surgical and post-surgical treatment strategies. Molecular testing for mutation hotspots, rearrangements, and gene expression using fine-needle aspiration specimens has become an effective diagnostic tool to more precisely select patients for an appropriate surgical procedure. Molecular testing surely would reduce the number of thyroidectomies done for benign nodules and tumors (16,17), and could guide determine the extent of initial surgical procedure (i.e., lobectomy versus total thyroidectomy). Beyond the driver mutations, it was also discovered that several individual key genes [CHEK2, ATM, and Telomerase reverse transcriptase (TERT)] and sets of functionally related genes (chromatin remodeling) with alterations or expression patterns in microRNA (miR-21 and miR-146b) define clinically-relevant subclasses and may contribute to loss of differentiation and tumor progression.

The IGCPTC study demonstrated striking signaling differences in RAS- and BRAFV600E-driven PTCs. In particular, BVL-PTCs signal preferentially through MAPK while RL-PTCs signal through both MAPK and PI3K. The relative simplicity of the PTC genome, with dominant mutually exclusive driving events, together with the large cohort and comprehensive data analyzed in this study have led to clearly dissect these signaling differences. The overreaching conclusion of the IGCPTC study was that RL-PTCs and BVL-PTCs are fundamentally different in their genetic, epigenetic, and proteomic profiles. Based on the strength of the multidimensional genomic findings, a clinico-pathologic reclassification of follicular-derived thyroid lesions would be imperative (15).

The impact of different genomic markers on outcomes opened a new field of controversy. Conflicting results with BRAF (+) tumors have been reported for the past 10 years. An increasing number of studies, which include meta-analyses, demonstrated an association between the BRAF status and aggressive tumor behavior. Other studies, however, have failed to confirm this data, and this has resulted in uncertainty about the prognostic value of BRAF mutations in PTC (18,19) It has become clear that multiple genetic alterations contribute to form an individual tumor phenotype and biology. A progressive genomic instability leads to first functional, then morphologic dedifferentiation. TERT promoter mutations have been observed in 5% to 25% of DTC, and it’s been reported that TERT promoter mutations contribute to aggressive behavior in DTC (20,21,22). These mutations have a significantly higher prevalence in aggressive thyroid tumor types such as widely invasive oncocytic carcinomas, poorly differentiated carcinomas, and anaplastic thyroid carcinomas (ATC) (23). Similarly, TP53 mutations are prevalent in advanced tumors with a higher recurrence (24). A co-occurrence of multiple mutations may define a more aggressive subgroup of DTC (25).

The next-generation sequencing technology allows high out-put genomic analysis. An innovative assay in thyroid cancer ThyroSeq® - was developed for targeted mutation detection by next generation sequencing technology in fine needle aspiration and tissue samples. ThyroSeq v.2 next generation sequencing panel offers simultaneous sequencing and detection in >1000 hotspots of 14 thyroid cancer-related genes and for 42 types of gene fusions known to occur in thyroid cancer (Table 3) (26). ThyroSeq is being increasingly used to further narrow the indeterminate category defined by cytology for thyroid nodules. From a surgical perspective, obviously this provides prognostic and predictive information as it relates to determination of surgical strategy. Both the genomic analysis technology and the data collection for the cancer genome atlas are rapidly developing. 

**Figure 2:**
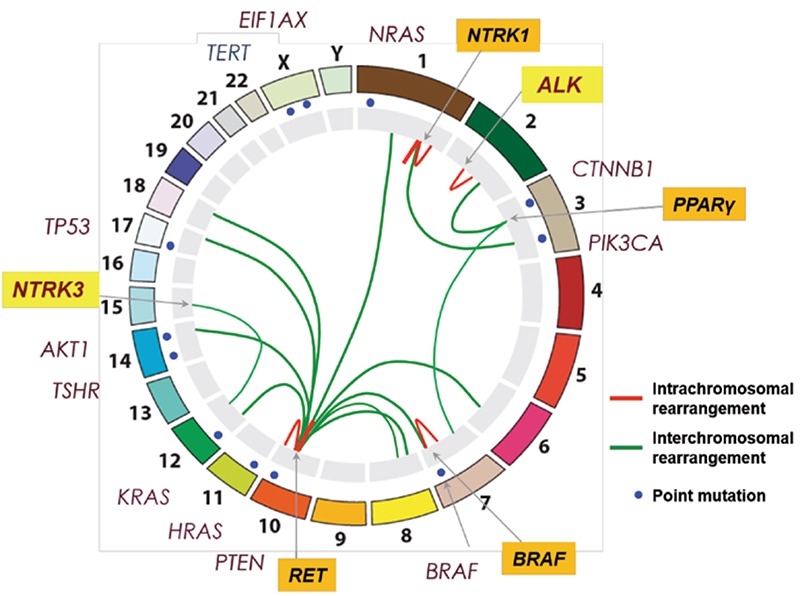
Main mutation mechanisms: Point mutations and chromosome rearrangements Adapted from Nikiforov YE, Diagnostic pathology and molecular genetics of the thyroid, 2009

Although we have sequenced the cancer genome extensively, and have identified a number of driver and passenger mutations, we are yet far from having a map of the full genomic alterations involved in the onco-pathogenesis of thyroid cancer (Figure 2). We are, though, capable of patterning the phenotypic expressions in terms of profiling. Individual tumor morphology, function and biology are compound results of mutational changes, and selective activation and/or deactivation of gene switches. Eventually, the cancer morphology, or architectural neo-differentiation will be understood and defined in terms of sets of genes that are expressed (transcribed) in neoplastic tissues. Perhaps the “papillary thyroid cancer” assignment based on morphology, its intricate variations, and perplexing functional and prognostic associations will be replaced by gene expression profiles, mathematical abstractions, with clear indications as to its biologic meaning. We will, though, continue to refer to the morphology as part of its complete profile, not a sole indicator of its prognosis and treatment options. This will also pave the road to rational treatment actions. The concept of risk stratification based on traditional parameters will soon vacate their role for clear molecular markers of non-invasive/focal, invasive/metastatic and systemic stages/phases of neoplastic disorder. 

Truly local/indolent tumors could easily be addressed with less invasive techniques such as Laser ablation and radiofrequency ablation, at the most by lobectomy. On the other hand when a potential for metastatic development is identified, regardless of the tumor size or patient’s age, the most aggressive approach including complete thyroidectomy (surgical total thyroidectomy followed by beta-knife ablation) will be employed. We will not feel inadequate to explain to an unfortunate patient why his/her small, supposed to be indolent tumor is taking a fatal course. We will not record those cases as statistical misfits. Patient-specific treatment protocols will prevail. A refined classification scheme based on genomics and its phenotypic expressions will accurately reflect the biologic differences between the different morphologic definitions we use today. Tumor differentiation/de-differentiation, and clinical behavior of an individual cancer will be defined by molecular markers, in addition to standard morpho-pathology. Empiricism in science of medicine and surgery has acquired a new method for testing the appropriate treatment for individual patients; that is molecular pathology, governed by genomics. The technology is present and rapidly evolving. The surgeons will determine the extent of interventions with molecular evidence and guidance.

The translation of cancer genomics data into clinical insights will elevate oncology to the next level and usher in a new era in understanding, diagnosing and treating cancer. The new evidence in “evidence-based medicine” is the genomic data.

In god we trust,

All others must bring data.

**William Edward Deming**

## Figures and Tables

**Table 1:**
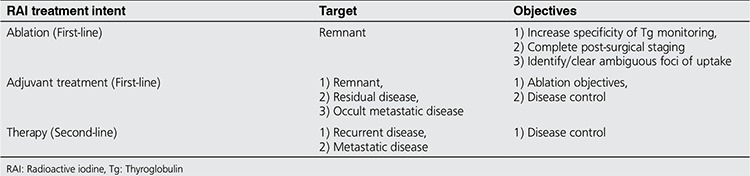
Radioactive iodine ablation objectives

**Table 2:**
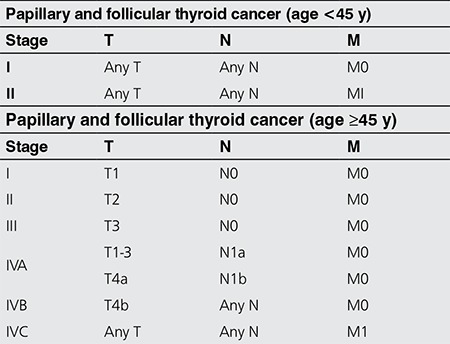
Staging of thyroid cancer

**Table 3:**
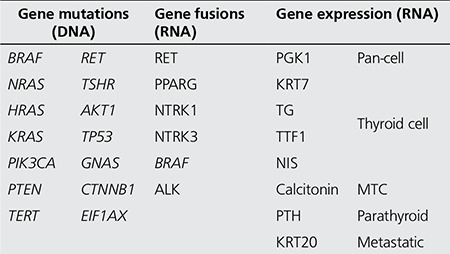
ThyroSeq v2 panel
